# Lesion evidence for a causal role of the insula in aversion to social inequity

**DOI:** 10.1093/scan/nsab098

**Published:** 2021-08-20

**Authors:** Felix Jan Nitsch, Hannah Strenger, Stefan Knecht, Bettina Studer

**Affiliations:** Comparative Psychology, Institute of Experimental Psychology, Heinrich-Heine-University Düsseldorf, Düsseldorf 40225, Germany; Department of Neurology, Mauritius Hospital Meerbusch, Meerbusch 40670, Germany; Department of Neurology, Mauritius Hospital Meerbusch, Meerbusch 40670, Germany; Institute of Clinical Neuroscience and Medical Psychology, Medical Faculty, Heinrich-Heine-University Düsseldorf, Düsseldorf 40225, Germany; Department of Neurology, Mauritius Hospital Meerbusch, Meerbusch 40670, Germany; Institute of Clinical Neuroscience and Medical Psychology, Medical Faculty, Heinrich-Heine-University Düsseldorf, Düsseldorf 40225, Germany

**Keywords:** inequity aversion, insula, computational modeling, social decision-making, value-based choices

## Abstract

Humans resist unequal distributions of goods in their social interactions, even if it requires foregoing personal gains. Functional neuroimaging studies implicate the insula in this aversion to social inequity and in fairness-related decisions, but a causal contribution has not yet been established. We compared the responses of 30 patients with lesions to the insula on a multiple-trial version of the one-shot Ultimatum Game, a neuroeconomic social exchange paradigm where a sum of money is split between two players, to those of 30 matched patients with brain injuries sparing the insula. Insula lesion patients accepted offers of an unequal disadvantageous split significantly more often than comparison lesion patients. Computational modeling confirmed that this difference in choice behavior was due to decreased aversion to disadvantageous inequity following insula damage, rather than due to increased decision noise or non-consideration of inequity. Our results provide novel evidence that the insula is causally involved in aversion to inequity and in value-based choices in the context of social interactions.

## Introduction

The distribution of goods is central to the makeup of human societies. Topics such as public health-care provision (like coronavirus disease-2019 vaccines), performance bonuses for managers and taxation rates are heatedly discussed. Also, our judgment of justifications for distribution and retribution policies depend upon our perception of fairness. Over the past decade, the importance of fairness in social decision-making has been extensively investigated in behavioral economics. One paradigm used for this purpose is the Ultimatum Game (UG; Güth *et al.*, 1982). In this two-player game, one player (‘the proposer’) has to split an amount of money between themselves and the other (‘the responder’), who can either accept the proposed split or reject it (in which case neither player gets any money). A wealth of studies using the UG has consistently found that responders reject offers that are perceived as unfair (see review by Güth and Kocher, 2014). Such behavior is costly, because it means forgoing one’s own payout, and constitutes a violation of classic economic models of rationality. It is thought to be at least partially driven by an affectively flavored aversion against inequity (‘inequity aversion’, e.g. Fehr and Schmidt, 1999; Oberliessen and Kalenscher, 2019).

Research in social neuroscience has started to elucidate how our brain processes inequity. Functional magnetic resonance imaging (fMRI) studies of the UG and other social decision-making paradigms point toward a strong involvement of the insula, a brain area also implicated in empathy and the appraisal of social norm violations (Molenberghs *et al.*, 2012; Grecucci *et al.*, 2013; Valk *et al.*, 2015; Lockwood, 2016; O’Connell *et al.*, 2019; Tholen *et al.*, 2020). In UG responders, the insula is more strongly activated during the processing of unfair than of fair offers (Sanfey, 2003; Corradi-Dell’Acqua *et al.*, 2016) and the degree of this activation difference is parametrically modulated by the emotional reappraisal of the proposer’s intentions (Grecucci *et al.*, 2013). Furthermore, increased insula activity positively predicts offer rejection decisions on the UG (Hollmann *et al.*, 2011; Guo *et al.*, 2013; Feng *et al.*, 2015), and effective connectivity between the anterior insula and the anterior midcingulate cortex was found to correlate with individuals’ reciprocity in a social interaction task (Shaw *et al.*, 2018). Some recent fMRI studies have also attributed functional activity patterns within subregions of the insula to emotional and cognitive processes that might drive inequity aversion. For instance, Gao *et al.* (2018) suggested that aversion to disadvantageous inequity (whether the subject receives less than their co-player) is primarily associated with emotion- and conflict-related processes recruiting the posterior insula (among other regions), whereas aversion to advantageous inequity results from mentalizing-related processes recruiting the anterior insula (and other structures). Based on a meta-analysis, Bellucci *et al.* (2018) argued that the dorsal anterior insula might mediate cognitive processes that generate expectancy for norm compliance, whereas the ventral anterior insula might mediate aversive feelings that generate motivation for norm enforcement.

Despite this rich functional neuroimaging data, investigations that would allow causal inferences concerning the involvement of the insula in inequity aversion and fairness-related social decision-making are still missing. The aim of the current study was to fill this gap of knowledge by studying patients with a focal lesion to the insula (*n* = 30). We compared the choices of insula lesion patients on an multiple-trial version of the one-shot UG (responder role) to a group of age-, gender- and functional impairment-matched patients with brain lesions sparing the insula (*n* = 30) and used a computational model to estimate patients’ aversion to disadvantageous inequity (Fehr and Schmidt, 1999) and choice sensitivity. Based on the aforementioned neuroimaging results, we predicted that aversion to disadvantageous inequity would be weakened or abolished after damage to the insula and that—as a result—insula lesion patients would accept more unequal offers than comparison lesion patients.

## Methods

### Participants and procedures

**Fig. 1.**
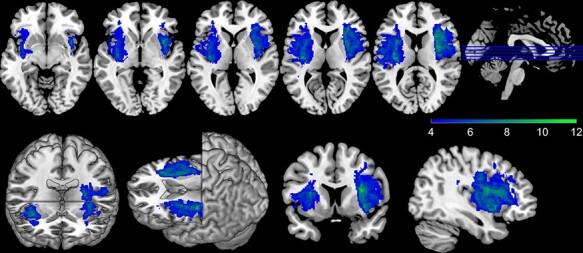
Lesion overlap in the insula lesion group. Visualization of the lesion overlap of the patients with damage to the insula (*n* = 30). The color bar indicates the number of overlapping cases at each voxel.

Thirty adult German-speaking stroke patients with lesions of the insula (‘insula lesion group’; *n* = 16 with damage to the right insula, *n* = 14 with damage to the left insula; see Figure 1) and 30 adult German-speaking stroke patients with lesions sparing the insula (‘comparison lesion group’) took part in this study. Patients in the comparison lesion group were matched to those in the insula lesion group on a one-to-one basis in gender, age and degree of functional impairment, quantified by the Barthel Index (Mahoney and Barthel, 1965; Lübke *et al.*, 2004; Table 1). This target sample size of *n* = 60 (*n* = 30 per group) allowed us to detect a large group effect (*f* = 0.40) in model-estimated inequity aversion (for details see section Computational Modelling) with a power of 1 − Beta = 0.861.

**Table 1.** Demographics, clinical background data and questionnaire scores

	Insula lesion group	Comparison lesion group	Group comparison	
	(*n *= 30)	(*n *= 30)	χ^2^/*T*	*P*
Gender (*n*, %)				
Female	14 (46.7)	14 (46.7)	0	1.000
Male	16 (53.3)	16 (53.3)		
Age (*M*, SEM)	71.87 (2.07)	71.90 (1.90)	−0.05	0.962
Barthel Index (*M*, SEM)	73.33 (4.22)	72.00 (3.71)	0.42	0.675
Diagnosis (*n*, %)				
Ischemic stroke	28 (93.3)	27 (90.0)	0.22	0.640
Hemorrhagic stroke	2 (6.7)	3 (10.0)		
Questionnaires (*M*, SEM)				
DASS-21	4.86 (0.70)	4.24 (0.85)	0.55	0.587
HADS-D	4.83 (0.75)	4.90 (0.66)	−0.06	0.951
AES	12.17 (1.52)	12.86 (1.70)	−0.27	0.791
AMI				
Total score	1.29 (0.10)	1.32 (0.08)	−0.27	0.786
Behavioral	6.34 (0.78)	6.41 (0.61)	−0.08	0.938
Social	9.62 (0.90)	10.14 (0.91)	−0.41	0.682
Emotional	7.17 (0.66)	7.21 (0.74)	−0.03	0.974

*Note:* DASS-21 = depression subscale of the Depression Anxiety Stress Scales (clinical cut-off ≥ 10), HADS-D = depression subscale of the Hospital Anxiety and Depression Scale (clinical cut-off ≥ 8), AES = Apathy Evaluation Scale (clinical cut-off ≥ 18), AMI = Apathy Motivation Index.

All patients were recruited during inpatient post-acute neurorehabilitation at the Mauritius Hospital Meerbusch. Exclusion criteria were severe cognitive impairment, aphasia and isolation due to multiresistant germs. For the control group, damage to the basal ganglia was also an exclusion criterion. In total, *n* = 2031 patients were screened for eligibility, *n* = 73 of which fulfilled the inclusion criteria for the insula lesion group and *n* = 201 of which fulfilled the inclusion criteria for the lesion comparison group (see Supplementary Figure S1 in Supplementary Material for a detailed description of the screening and recruitment process).

Patients underwent one or two behavioral testing sessions, depending on attention and fatigue spans, which lasted approximately 60 min in total. They completed the UG and an effort-based decision-making task (data not reported here). Additionally, they completed four self-report questionnaires on symptoms of depression and apathy: the Apathy Evaluation Scale (Marin *et al.*, 1991; Lueken *et al.*, 2006), the Apathy Motivation Index (Ang *et al.*, 2017), the depression subscale of the Hospital Anxiety and Depression Scale (Zigmond and Snaith, 1983; Herrmann *et al.*, 1995), and the depression subscale of the 21-item version of the Depression Anxiety Stress Scales (Lovibond and Lovibond, 1995; Antony *et al.*, 1998; Table 1).

### Ultimatum Game

**Fig. 2.**
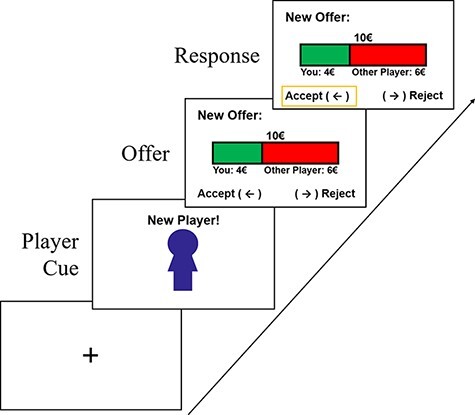
UG. An exemplary trial of the UG is shown. First, a cue reminding patients that they will be playing against a new opponent was presented. Next, patients received an offer from this anonymized co-player on how the pot of €10 will be split between them. Then they indicated whether they wanted to accept this offer (in which case both players receive the attributed amounts) or reject it (in which case neither gets money and the pot decays).

We used a multiple-trial version of the one-shot UG, where participants played against a new, gender-matched, anonymized virtual opponent on each trial. Patients were told that they would be connected to an online platform and play against real anonymized co-players. They were further told that they would be randomly assigned to either the proposer or responder role at the beginning of the game and keep this role throughout the game. In truth, all patients were assigned the responder role, co-players were computer simulated and presented offers were predetermined. The instructions also emphasized that patients would play with a new person in each round. This design allowed us to repeatedly sample their responses to each offer while avoiding meta-cognitive influences that may occur in repeated interactions with the same co-player and attractiveness, and racial and other implicit biases that may occur when playing against non-anonymized opponents (Solnick and Schweitzer, 1999; Mendoza *et al.*, 2014). The amount to be split was €10 in all cases, the offer size varied from €0:€10 (highly disadvantageous) to €5:€5 (equal split), in 1-euro steps, across trials. In each trial, patients were presented with one of these offers, with the amount allocated to them and the amount kept by the co-player, indicated both numerically and visually. The patients then decided whether to accept or reject the offer and indicated their decision through pressing a corresponding button on the keyboard (see Figure 2). Patients completed 55 trials, which entailed five repetitions of the €0:€10 offer and 10 repetitions of each other offer (i.e. €1:€9, €2:€8, €3:€7, €4:€6 and €5:€5). Prior to the start, task comprehension was confirmed through two qualitative questions. At the end of the task, one trial was selected for effective payout based on patient’s recorded choice. The task was self-paced; on average, patients took approximately 15–20 min to complete it. Mean acceptance rates for each offer acted as a primary behavioral outcome measure.

### Computational modeling

To decompose the processes underlying patients’ responses on the UG, we fitted a reduced Fehr–Schmidt inequity aversion model to their data using maximum-likelihood estimation:

$${\rm{U}}\left( {{\rm{offer}}} \right){\rm{ = V\_Self - i^*}}\left( {{\rm{V\_Other - V\_Self}}} \right)$$

where *U*(offer) is the utility of the offer from the perspective of the responder, V_Self is the amount allocated to the responder, V_Other is the amount allocated to the proposer (i.e. the co-player) and the estimated parameter *i* reflects the degree of aversion to disadvantageous inequity (restricted to vary between zero and two^1^). Trial-by-trial estimates of *U* were transformed into probability of offer acceptance using:

$${\rm{p}}\left( {{\rm{accept}}} \right){\rm{ = logistic}}\left( {{\rm{U,mu}}} \right)$$

where mu is an inverse temperature parameter that characterizes the choice consistency or lack of stochasticity (restricted to be larger than zero). To assess the general validity of our model, we compared it to a dummy model disregarding an influence of the Offer Type and assuming a fixed acceptance rate per group (*p* = *c*) and a prosocial model given by:

$${\rm{U}}\left( {{\rm{offer}}} \right){\rm{ = V\_Self + w^*V\_Other}}$$

where *w* reflects the degree of prosocial preference that determines how much the gain of others is weighed for one’s own utility (restricted to vary between zero and two).

Furthermore, we compared the predictions of our inequity aversion model to those of a self-gain-only model:

$${\rm{U}}\left( {{\rm{offer}}} \right){\rm{ = eta^*V\_Self}}$$

where eta is a non-negative scaling parameter. For offers with a V_self > 0, the predictions of this model align with that of the inequity aversion model. However, in the case of a €0:€10 offer, the inequity aversion model postulates a negative utility and thus predicts *p*(accept) < 0.5, whereas this self-gain-only model postulates *U*(offer) = 0 and predicts a *p*(accept) ≈ 0.5.

### Statistical analysis of behavioral data and model parameters

All statistical analyses were conducted using R (R Core Team, 2020), particularly the stats4 and lme4 (Bates *et al.*, 2015) packages. The significance level was set to alpha = 0.05 for all analyses. In a first step, we tested for differences in the accept/reject choices of the insula lesion and the control lesion group, with a logit-binomial generalized linear mixed effects model with the lesion group, offer (six levels) and their interaction, as well as total lesion volume as fixed effects and a subject-level random intercept. Follow-up *post*  *hoc* comparisons were conducted with Bonferroni correction applied to *P*-values. Next, we compared the parameters of our computational models (inequity aversion model, prosocial model and dummy model) between the two groups, while statistically controlling for total lesion volume, using analyses of covariance. To differentiate the inequity aversion model from a self-gain-only model in the choice domain, we ran a one-sided binomial test on observed acceptance rates of the €0:€10 offer. Graphical outputs were created with the ggplot2 (Wickham, 2011) package. An exploratory analysis of choice times is reported in Supplementary Material (see Supplementary Figure S3).

### Neuroanatomical analysis

Patients’ lesions were traced on a structural T1- or T2-weighted MRI (*n* = 58) or CT (*n* = 2) scan using the MRIcron software (Rorden and Brett, 2000) and the SPM toolbox Clusterize (De Haan *et al.*, 2015). Next, structural scans and lesion traces were normalized to an MNI305 template using the Clinical Toolbox (Rorden *et al.*, 2012) in SPM12, using lesion-masked (Brett *et al.*, 2001) or enantiomorphic (Nachev *et al.*, 2008) normalization. The total volume of brain damage was extracted for each patient using MRIcron. Since average total brain damage was significantly larger in insula lesion group (*M* = 44.55 cm^3^, SEM = 10.88) than in the comparison lesion group (*M* = 12.29 cm^3^, SEM = 4.92), total lesion volume was statistically controlled for in all group comparisons of behavioral data and model parameters. For patients in the insula group, lesion volume within the grey matter of the insular cortex (Automated anatomical labelling (AAL) atlas regions 29 and 30) was further extracted, in order to test whether model-estimated inequity aversion was negatively correlated with the extent of insula damage (controlled for total volume of brain damage outside the insula). Exploratory analyses assessing potential effects of insula lesion laterality and of insula subregions are reported in Supplementary Material (see Supplementary Figures S4 and S5). Finally, a whole-brain voxel-based lesion-behavior mapping analysis conducted in NiiStat tested whether model-estimated inequity aversion and choice sensitivity was predicted by damage of particular voxels in the brain. Voxels affected in at least 10% of patients across both groups were considered (*n* = 4007), and Bonferroni correction was applied.

## Results

### Behavioral analysis

**Fig. 3.**
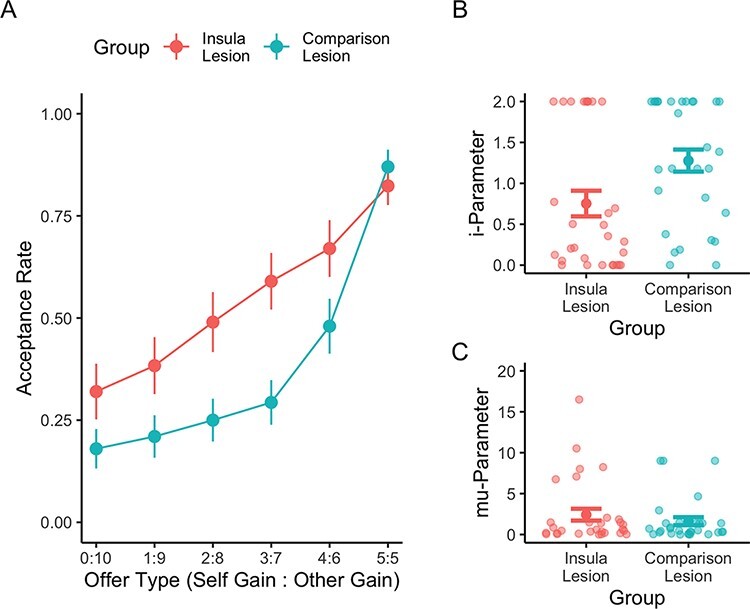
Responses on the UG and model-estimated inequity aversion. Panel A shows the average acceptance rates per group and offer. Insula lesion patients showed significantly higher acceptance rates than control patients for disadvantageous offers but not for the equal-split offer. Panel B shows the average estimate and the complete distribution of the inequity aversion parameter *i* per group. In alignment with the behavioral results, insula patients showed a significantly lower inequity aversion than control patients. Panel C shows the average estimate and the complete distribution of the choice consistency parameter *m* per group, for which there was no significant group difference. Error bars indicate the standard error of the mean.

Mean acceptance rates for each offer level in the two groups are provided in Table 2 and Figure 3A. The analysis of this main behavioral outcome found a significant interaction effect of lesion group and offer (*z* = 2.548, *P* = 0.011, main effect of offer). Follow-up separate comparisons of the (pooled) acceptance rates of disadvantageous offers and the acceptance rates of the equal-split offer showed that insula lesion patients accepted disadvantageous offers significantly more often than comparison lesion patients (*W* = 14 576, *P*_corr_ < 0.001, *r* = 0.57), whereas acceptance rates of the equal split offer did not differ between the groups (*W* = 412, *P*_corr_ = 1.00). Furthermore, a significant effect of total lesion volume on the acceptance rates was found (*z* = 2.742, *P* = 0.006), indicating that a larger brain damage was associated with higher acceptance rates.

**Table 2.** Mean acceptance rates for each offer level

	Insula group		Control group	
Offer (self: other)	*M*	SEM	*M*	SEM
€0:€10	0.320	0.068	0.180	0.048
€1:€9	0.383	0.070	0.210	0.052
€2:€8	0.490	0.073	0.250	0.052
€3:€7	0.590	0.069	0.293	0.055
€4:€6	0.670	0.070	0.480	0.067
€5:€5	0.823	0.057	0.870	0.042

### Model-based results

The inequity aversion model provided a better description of the data than the prosocial and dummy models for most patients (*n* = 16 of the insula lesion group and *n* = 23 of the comparison lesion group) according to both the Akaike Information Criterion (AIC) and the Bayesian Information Criterion (BIC; see Supplementary Figure S2 in Supplementary Material). Acceptance rates for the €0:€10 offer were significantly lower than 0.5 (*M*_InsulaGroup_ = 0.32. s.d. = 0.374, *P* < 0.001, *M*_ComparisonGroup_ = 0.18, s.d. = 0.264, *P* < 0.001), in line with the prediction of the inequity aversion model, but not that of the self-gain-only model. In summary, patients’ choices were best explained by the inequity aversion model.

Average inequity aversion (i) and choice consistency (mu) parameters from the inequity aversion model are displayed in Figure 3B–C. As predicted, inequity aversion was significantly lower in the insula lesion group than in the comparison lesion group (*F* = 4.192, *P* = 0.045, ηp^2^ = 0.07), whereas choice consistency did not differ significantly (*F* = 1.284, *P* = 0.262). Given that *i* and mu were not normally distributed, we repeated the group comparison with nonparametric Mann–Whitney *U* tests. Results remain qualitatively unchanged (see Supplementary Material for details). The two parameters were not correlated significantly (*r* = −0.220, *P* = 0.091; rho = −0.197, *P* = 0.132).

The extent of insula damage negatively predicted superiority of the inequity aversion model over the null model in the insula lesion group (Wald χ^2^ = 5.503, *P* = 0.019, odds ratio [OR] = 0.788, 95% confidence interval [CI] = [0.646, 0.962]). However, this relationship was rendered statistically non-significant when controlling for volume of total brain damage outside the insula (χ^2^ = 2.300, *P* = 0.129, OR = 0.827, 95% CI = [0.648, 1.057]). The exploratory voxel-based whole-brain analyses yielded no voxels whose damage significantly predicted inequity aversion or choice consistency across the two groups.

## Discussion

The current study tested whether the insula plays a causal role in inequity aversion and fairness-related social decision-making. Choices of patients with insula lesions on a multiple-trial version of the one-shot UG (responder role) were compared to those of age-, gender- and general-impairment-matched patients with lesions sparing the insula. We found that insula lesion patients accepted unequal offers significantly more often and had reduced model-estimated aversion to disadvantageous inequity than comparison lesion patients.

Our results provide novel evidence that the insula is causally involved in value-based choices in the context of social inequity. The observed lower tendency to reject unfair offers is consistent with neuroimaging studies showing stronger activation of the insula in response to unfair offers (Sanfey, 2003; Hollmann *et al.*, 2011; Guo *et al.*, 2013; Feng *et al.*, 2015; Corradi-Dell’Acqua *et al.*, 2016), but critically extends this previous data by showing that damage to this structure manifests in altered behavior. Our modeling results further confirm that the decreased rejection of unequal offers by our insula lesion patients was a direct consequence of reduced inequity aversion, rather than increased noise in valuation (captured by the choice sensitivity parameter) or an indifference toward or lack of processing of the gains of others (captured by the self-gain-only model). To our knowledge, only one other study (Gu *et al.*, 2015) has administered the UG to patients with insula lesions and found no differences in the acceptance rates of these patients *vs* healthy or lesion controls. However, their insula lesion group consisted of only seven patients and was therefore heavily underpowered for detecting an effect akin to the one we observed (*post*  *hoc* power = 0.269 for an effect of *r* = 0.57). Using a dynamic Rescorla–Wagner norm adaptation model, Gu and colleagues did find an increased sensitivity to norm violations of UG proposers in their insula lesion patients. Computationally, this parameter of their model is similar to the inequity aversion parameter of the classic Fehr–Schmidt model, which tentatively suggests an opposite effect as in our data. However, given that overt behavior in that study was insensitive to insula lesions, it is difficult to judge the comparability of these findings with those of the current study.

Previous neuropsychological studies have implicated the insula in value-based decision-making in non-social contexts. On laboratory tasks of decision-making under risk, insensitivity to differences in expected value between choice options (Weller *et al.*, 2009) and impaired adjustment of betting choices to the odds of winning (Clark *et al.*, 2008) were observed. Another study (Shiv *et al.*, 2005) found reduced loss aversion on an investment task in patients with insula damage. Finally, Clark *et al.* (2014) showed that insula lesion patients are less susceptible to cognitive distortions during gambling. The authors of the latter two studies and others (e.g. Singer *et al.*, 2009; Markett *et al.*, 2016; Von Siebenthal *et al.*, 2016), have argued that these abnormalities in value-based decision-making following insula lesions might be related to the insula’s involvement in interoception and the processing of peripheral emotion signals. While we have not collected affect ratings, this hypothesis is broadly consistent with our behavioral findings, as inequity aversion and rejections on the UG have been postulated to be fueled by an emotional response to (perceived) unfairness (c.f. Oberliessen and Kalenscher, 2019). Perhaps paradoxically, the reduced rejection of unequal offers observed in our insula patients would be more rational according to some classic models of economic decision-making. However, inequity aversion is thought to protect individuals from exploitation and allow for long-term cooperation with non-kin (Brosnan, 2011; Brosnan and de Waal, 2014). Neuroimaging studies have also implicated the insula in the modulation of social affect by perceived intentions of others (Grecucci *et al.*, 2013), conflict-related processes (Gao *et al.*, 2018) and vicarious experience (related to empathy; Lockwood, 2016). Together, these findings implicate the insula in the processing of and response to others’ actions and intentions.

We note three limitations to the current study. The first is that the insula obviously acts not in isolation, but rather within an interconnected network. For instance, Touroutoglou *et al.* (2012) found that two distinct networks are anchored in the right anterior insula: a network related to the intensity of affective experience and a network associated with attention and performance speed. In the context of social decision-making, a recent study by Shaw *et al.* (2018) identified effective connectivity between the anterior insula and the anterior midcingulate cortex as a neural correlate of individuals’ reciprocity. Our data do not allow examining such network interactions, but future studies might aim to investigate how insula damage affects network activity during social and non-social value-based decision-making. A second limitation is that damage in the insula lesion group often encroached into the dorsal striatum (situated within the same vascular territory) and, while this represents one of the largest studies of insula damage in the literature, we had limited power to statistically control for a potential contribution of striatal pathology to the observed abnormalities in UG choices. However, single-case analysis indicated that insula lesion patients without striatal involvement were among those with the most strongly attenuated inequity aversion (see Supplementary Figure S6 in Supplementary Material), speaking against striatal damage underlying the results observed in the insular group. A third constraint is that our task design only included inequity that was disadvantageous to the assessed individuals. Future work may test if the insula is also involved in the perception of and reaction to advantageous social inequity.

## Conclusions

We here demonstrate that aversion to disadvantageous inequity is systematically hampered in patients with insula lesions, whereas choice consistency was unchanged. This presents novel evidence that the insula is causally involved in social decision-making in general, and in the processing and response to social inequity, in specific.

## Supplementary Material

nsab098_Supp

## Data Availability

The anonymized dataset and analysis code of the current study are available in the Open Science Framework (OSF) repository, https://osf.io/b9fhc/?view_only=02512ecf560545f49b5ee8ece228a275.

## Footnotes

1 We restricted the parameter spaces for *i* (in case of Fehr–Schmidt inequity aversion model) and *w* (in case of the prosocial model) to avoid inflation of parameter estimates for subjects that are overall less well-described by the model. Furthermore, a larger parameter space (*i* and *w* < 10) improved the fit of the models only marginally according to AIC (Mdelta = 2.453) and BIC (Mdelta = 2.043).

## References

[R1] Ang Y.-S., Lockwood P., Apps M.A., et al. (2017). Distinct subtypes of apathy revealed by the apathy motivation index. *PLoS**One*, 12, e0169938. 10.1371/journal.pone.0169938 PMC5226790 28076387

[R2] Antony M.M., Bieling P.J., Cox B.J., et al. (1998). Psychometric properties of the 42-item and 21-item versions of the Depression Anxiety Stress Scales in clinical groups and a community sample. *Psychological Assessment*, 10, 176.

[R3] Bates D., Mächler M., Bolker B., et al. (2015). Fitting linear mixed-effects models using lme4. *Journal of Statistical Software*, 67, 1–48.

[R4] Bellucci G., Feng C., Camilleri J., et al. (2018). The role of the anterior insula in social norm compliance and enforcement: evidence from coordinate-based and functional connectivity meta-analyses. *Neuroscience and Biobehavioral Reviews*, 92, 378–89. 29958872 10.1016/j.neubiorev.2018.06.024

[R5] Brett M., Leff A.P., Rorden C., Ashburner J. (2001). Spatial normalization of brain images with focal lesions using cost function masking. *NeuroImage*, 14, 486–500. 11467921 10.1006/nimg.2001.0845

[R6] Brosnan S.F. (2011). A hypothesis of the co-evolution of cooperation and responses to inequity. *Frontiers in Neuroscience*, 5, 43. 10.3389/fnins.2011.00043 PMC3077916 21519380

[R7] Brosnan S.F., de Waal F.B. (2014). Evolution of responses to (un)fairness. *Science*, 346, 1–9. 10.1126/science.1251776 PMC4451566 25324394

[R8] Clark L., Bechara A., Damasio H., et al. (2008). Differential effects of insular and ventromedial prefrontal cortex lesions on risky decision-making. *Brain*, 131, 1311–22. 18390562 10.1093/brain/awn066 PMC2367692

[R9] Clark L., Studer B., Bruss J., et al. (2014). Damage to insula abolishes cognitive distortions during simulated gambling. *Proceedings of the National Academy of Sciences*, 111, 6098–103. 10.1073/pnas.1322295111 PMC4000793 24711387

[R10] Corradi-Dell’Acqua C., Tusche A., Vuilleumier P., et al. (2016). Cross-modal representations of first-hand and vicarious pain, disgust and fairness in insular and cingulate cortex. *Nature Communications*, 7, 10904. 10.1038/ncomms10904 PMC4802033 26988654

[R11] De Haan B., Clas P., Juenger H., et al. (2015). Fast semi-automated lesion demarcation in stroke. *NeuroImage: Clinical*, 9, 69–74. 26413473 10.1016/j.nicl.2015.06.013 PMC4543214

[R12] Fehr E., Schmidt K.M. (1999). A theory of fairness, competition, and cooperation. *The Quarterly Journal of Economics*, 114, 817–68.

[R13] Feng C., Luo Y.-J., Krueger F. (2015). Neural signatures of fairness-related normative decision making in the ultimatum game: a coordinate-based meta-analysis: neural signatures of decision making in UG. *Human Brain Mapping*, 36, 591–602. 25327760 10.1002/hbm.22649 PMC6869807

[R14] Gao X., Yu H., Sáez I., et al. (2018). Distinguishing neural correlates of context-dependent advantageous-and disadvantageous-inequity aversion. *Proceedings of the National Academy of Sciences*, 115, E7680–89. 10.1073/pnas.1802523115 PMC6099874 30061413

[R15] Grecucci A., Giorgetta C., van’t Wout M., et al. (2013). Reappraising the ultimatum: an fMRI study of emotion regulation and decision making. *Cerebral Cortex*, 23, 399–410. 22368088 10.1093/cercor/bhs028

[R16] Gu X., Wang X., Hula A., et al. (2015). Necessary, yet dissociable contributions of the insular and ventromedial prefrontal cortices to norm adaptation: computational and lesion evidence in humans. *The Journal of Neuroscience*, 35, 467–73. 25589742 10.1523/JNEUROSCI.2906-14.2015 PMC4293403

[R17] Guo X., Zheng L., Zhu L., et al. (2013). Increased neural responses to unfairness in a loss context. *NeuroImage*, 77, 246–53. 23562770 10.1016/j.neuroimage.2013.03.048

[R18] Güth W., Schmittberger R., Schwarze B. (1982). An experimental analysis of ultimatum bargaining. *Journal of Economic Behavior and Organization*, 3, 367–88.

[R19] Güth W., Kocher M.G. (2014). More than thirty years of ultimatum bargaining experiments: motives, variations, and a survey of the recent literature. *Journal of Economic Behavior and Organization*, 108, 396–409.

[R20] Herrmann C., Buss U., Snaith R.P. (1995). *HADS-D Hospital Anxiety and Depression scale–Deutsche Version*, Bern: Huber.

[R21] Hollmann M., Rieger J.W., Baecke S., et al. (2011). Predicting decisions in human social interactions using real-time fMRI and pattern classification P. Villoslada (ed). *PLoS One*, 6, e25304. 10.1371/journal.pone.0025304 PMC3189203 22003388

[R22] Lockwood P.L. (2016). The anatomy of empathy: vicarious experience and disorders of social cognition. *Behavioural Brain Research*, 311, 255–66. 27235714 10.1016/j.bbr.2016.05.048 PMC4942880

[R23] Lovibond S.H., Lovibond P.F. (1995). *Manual for the Depression Anxiety Stress Scales*, Sydney: Psychology Foundation Australia.

[R24] Lübke N., Meinck M., von Renteln–Kruse W. (2004). Der Barthel–Index in der Geriatrie. Eine Kontextanalyse zum Hamburger Einstufungsmanual. *Zeitschrift Für Gerontologie Und Geriatrie*, 37, 316–26. 15338161 10.1007/s00391-004-0233-2

[R25] Lueken U., Seidl U., Schwarz M., et al. (2006). Psychometric properties of a German version of the apathy evaluation scale. *Fortschritte der Neurologie-Psychiatrie*, 74, 714–22. 17167730 10.1055/s-2006-932164

[R26] Mahoney F.I., Barthel D.W. (1965). Functional evaluation: the Barthel Index: a simple index of independence useful in scoring improvement in the rehabilitation of the chronically ill. *Maryland State Medical Journal*, 14, 61–5. 14258950

[R27] Marin R.S., Biedrzycki R.C., Firinciogullari S. (1991). Reliability and validity of the apathy evaluation scale. *Psychiatry Research*, 38, 143–62. 1754629 10.1016/0165-1781(91)90040-v

[R28] Markett S., Heeren G., Montag C., et al. (2016). Loss aversion is associated with bilateral insula volume. A voxel based morphometry study. *Neuroscience Letters*, 619, 172–6. 27012426 10.1016/j.neulet.2016.03.029

[R29] Mendoza S.A., Lane S.P., Amodio D.M. (2014). For members only: ingroup punishment of fairness norm violations in the ultimatum game. *Social Psychological and Personality Science*, 5, 662–70.

[R30] Molenberghs P., Cunnington R., Mattingley J.B. (2012). Brain regions with mirror properties: a meta-analysis of 125 human fMRI studies. *Neuroscience and Biobehavioral Reviews*, 36, 341–9. 21782846 10.1016/j.neubiorev.2011.07.004

[R31] Nachev P., Coulthard E., Jäger H.R., et al. (2008). Enantiomorphic normalization of focally lesioned brains. *NeuroImage*, 39, 1215–26. 18023365 10.1016/j.neuroimage.2007.10.002 PMC2658465

[R32] O’Connell K., Brethel-Haurwitz K.M., Rhoads S.A., et al. (2019). Increased similarity of neural responses to experienced and empathic distress in costly altruism. *Scientific Reports*, 9, 1–11. 31341206 10.1038/s41598-019-47196-3 PMC6656917

[R33] Oberliessen L., Kalenscher T. (2019). Social and non-social mechanisms of inequity aversion in non-human animals. *Frontiers in Behavioral Neuroscience*, 13, 133. 10.3389/fnbeh.2019.00133 PMC6598742 31293399

[R34] R Core Team . (2020). *R: A Language and Environment for Statistical Computing*. Vienna, Austria.

[R35] Rorden C., Bonilha L., Fridriksson J., et al. (2012). Age-specific CT and MRI templates for spatial normalization. *NeuroImage*, 61, 957–65. 22440645 10.1016/j.neuroimage.2012.03.020 PMC3376197

[R36] Rorden C., Brett M. (2000). Stereotaxic display of brain lesions. *Behavioural Neurology*, 12, 191–200. 11568431 10.1155/2000/421719

[R37] Sanfey A.G. (2003). The neural basis of economic decision-making in the Ultimatum Game. *Science*, 300, 1755–8. 12805551 10.1126/science.1082976

[R38] Shaw D.J., Czekóová K., Staněk R., et al. (2018). A dual-fMRI investigation of the iterated Ultimatum Game reveals that reciprocal behaviour is associated with neural alignment. *Scientific Reports*, 8, 10896. 10.1038/s41598-018-29233-9 PMC6051991 30022087

[R39] Shiv B., Loewenstein G., Bechara A., et al. (2005). Investment behavior and the negative side of emotion. *Psychological Science*, 16, 435–9. 15943668 10.1111/j.0956-7976.2005.01553.x

[R40] Singer T., Critchley H.D., Preuschoff K. (2009). A common role of insula in feelings, empathy and uncertainty. *Trends in Cognitive Sciences*, 13, 334–40. 19643659 10.1016/j.tics.2009.05.001

[R41] Solnick S.J., Schweitzer M.E. (1999). The influence of physical attractiveness and gender on ultimatum game decisions. *Organizational Behavior and Human Decision Processes*, 79, 199–215. 10471361 10.1006/obhd.1999.2843

[R42] Tholen M.G., Trautwein F., Böckler A., et al. (2020). Functional magnetic resonance imaging (fMRI) item analysis of empathy and theory of mind. *Human Brain Mapping*, 41, 2611–28. 32115820 10.1002/hbm.24966 PMC7294056

[R43] Touroutoglou A., Hollenbeck M., Dickerson B.C., et al. (2012). Dissociable large-scale networks anchored in the right anterior insula subserve affective experience and attention. *NeuroImage*, 60, 1947–58. 22361166 10.1016/j.neuroimage.2012.02.012 PMC3345941

[R44] Valk S.L., Bernhardt B.C., Böckler A., et al. (2015). Divergent network substrates of individual differences in empathy and mentalizing. In: 21st Annual Meeting of the Organization for Human Brain Mapping (OHBM). Honolulu: OHBM, 256.

[R45] von Siebenthal Z., Boucher O., Rouleau I., et al. (2017). Decision-making impairments following insular and medial temporal lobe resection for drug-resistant epilepsy. *Social Cognitive and Affective Neuroscience*, 12, 128–37. 27798255 10.1093/scan/nsw152 PMC5390706

[R46] Weller J.A., Levin I.P., Shiv B., et al. (2009). The effects of insula damage on decision-making for risky gains and losses. *Social Neuroscience*, 4, 347–58. 19466680 10.1080/17470910902934400

[R47] Wickham H. (2011). ggplot2: ggplot2. *Wiley Interdisciplinary Reviews: Computational Statistics*, 3, 180–5.

[R48] Zigmond A.S., Snaith R.P. (1983). The hospital anxiety and depression scale. *Acta Psychiatrica Scandinavica*, 67, 361–70. 6880820 10.1111/j.1600-0447.1983.tb09716.x

