# Supplementary material for: Lesion evidence for a causal role of the insula in aversion to social inequity
Source: Soc Cogn Affect Neurosci. 2021 Aug 20;17(3):266–72. doi: 10.1093/scan/nsab098 (PMC8881633; doi:10.1093/scan/nsab098)
Supplement: nsab098_Supp [file nsab098_supp.zip › scan-21-062-File007.docx]

# Supplementary Material for

# Lesion evidence for a causal role of the insula in aversion to social inequity

by

Authors: Felix Jan Nitsch, Hannah Strenger, Stefan Knecht, & Bettina Studer

**Table S1: Neuropsychological Screening**

|  | % impaired | *n* | χ^2^ | *p* |
| --- | --- | --- | --- | --- |
| Orientation |  |  |  |  |
| insula lesion group | 21.43 | 14 | .05 | .818 |
| comparison lesion group | 25 | 16 |  |  |
|  |  |  |  |  |
| Attention |  |  |  |  |
| insula lesion group | 93.75 | 16 | 4.54 | .033* |
| comparison lesion group | 61.54 | 13 |  |  |
|  |  |  |  |  |
| Executive functions |  |  |  |  |
| insula lesion group | 44.44 | 9 | .02 | .899 |
| comparison lesion group | 41.67 | 12 |  |  |
|  |  |  |  |  |
| Neglect or hemianopia |  |  |  |  |
| insula lesion group | 33.33 | 15 | 0 | 1 |
| comparison lesion group | 33.33 | 15 |  |  |
|  |  |  |  |  |
| Aphasia |  |  |  |  |
| insula lesion group | 11.11 | 18 | .31 | .581 |
| comparison lesion group | 5.88 | 17 |  |  |
|  |  |  |  |  |
| Verbal memory |  |  |  |  |
| insula lesion group | 41.67 | 12 | .67 | .414 |
| comparison lesion group | 58.33 | 12 |  |  |
|  |  |  |  |  |
| Non-verbal memory |  |  |  |  |
| insula lesion group | 28.57 | 14 | .07 | .793 |
| comparison lesion group | 33.33 | 12 |  |  |

*Note.* 19 insula patients and 18 control patients were assessed by a neuropsychologist, however not all listed cognitive domains were tested in every patient.

**
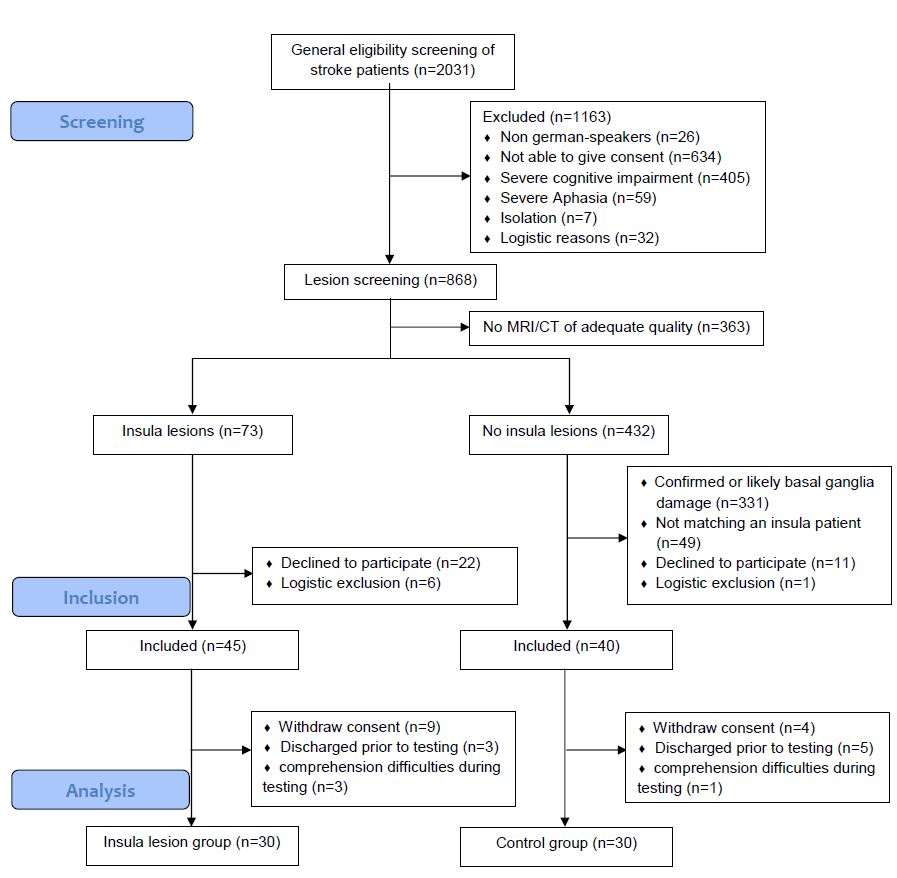
**

**Figure S1: Screening and recruitment**


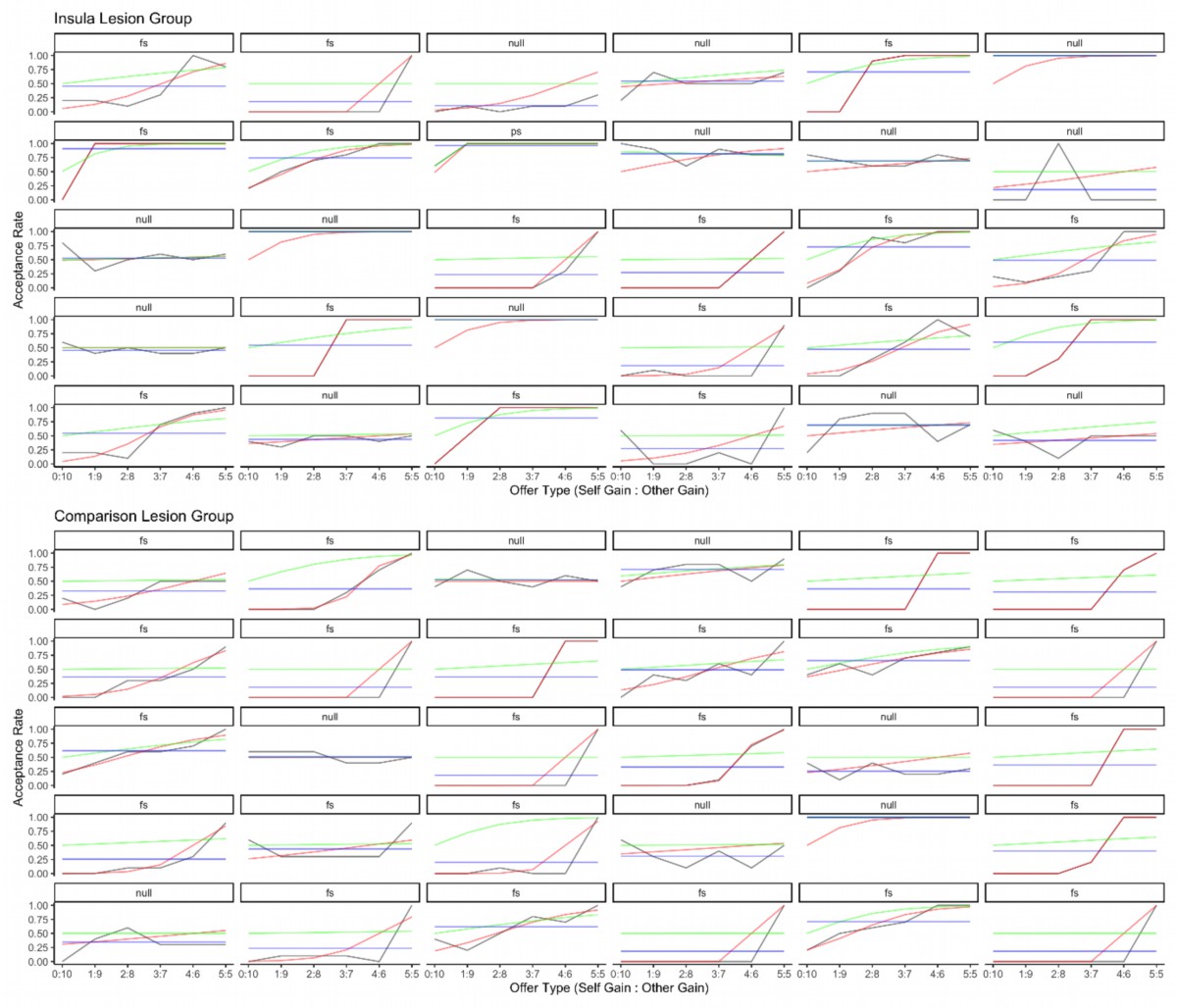


**Figure S2: Subject-level acceptance rates and model comparisons**

The top panel shows the subjects of the insula lesion group, the bottom panel shows the subjects of the comparison lesion group. In each panel, black lines indicate the individual patient’s recorded acceptance rates, red lines indicate predictions from Fehr-Schmidt style model, blue lines indicate predictions from dummy model, and green lines indicate predictions from prosocial model. Boxes indicate which model provided the best description of the data according to Akaike Information Criterion (fs = Fehr-Schmidt style model, null = null model, ps = prosocial model). The Fehr-Schmidt style model was favoured for most subjects.

**Supplementary analysis of modeling results**

Our primary analysis compared model-estimated inequity aversion (i) and choice consistency (mu) between the insula lesion and comparison lesion groups with an ANCOVA model that could statistically control for the total volume of brain damage. Given that the distributions of these model-estimated parameters deviated from the normal distribution (i: skewness = 0.088, kurtosis = -1.747, mu: skewness = 2.354, Kurtosis = 5.574), we repeated the group comparison with non-parametric Mann-Whitney-U tests (disregarding total lesion volume). Results remained qualitatively unchanged. Inequity aversion was significantly lower in the insula lesion group than in the control lesion group (W = 592, p = .036), whereas choice consistency did not differ significantly (W = 415, p = .605).

As a further supplementary robustness check of our computational modeling results, we also repeated the ANCOVA described in the main manuscript for only the subset of subjects that were best described by the inequity aversion model (n=16 insula lesion patients, and n=23 comparison patients; see Figure S2). Inequity aversion was again lower in the insula lesion group than in the control lesion group, albeit only on trend level (F(1,36) = 3.319, p = .077). We note that due to the unbalanced sample sizes in each group of this subset, this test had only a power of 1-Beta $\approx$ 0.33 to detect a medium-sized effect. Choice consistency did again not differ significantly between the two groups (F(1,36) = 2.587, p =.116).

**Exploratory analyses of decision times**

As noted in the main manuscript, the Fehr-Schmidt inequity aversion model can be dissociated from a (more basic) self-gain-only model on trials where the offer was €0:€10. In this case, the two models make divergent predictions regarding the utility of the offer and in consequence the accept likelihood. In the main manuscript, we describe how observed accept probabilities were more in line with the inequity aversion model. As a further exploratory check, we investigated patients’ decision times in these trials. Neither utility model makes direct predictions regarding response times, however assuming that response times are given by a decreasing function of subjective utility of the offer and random noise, we reasoned that the inequity-aversion model predicts longer response times for accept compared to reject decisions for the €0:€10 offer (given i > 0), whereas the self-gain-only model predicts equal response times for accept and reject decisions to this offer. Observed reaction times are displayed in Figure S3. In the insula lesion group, observed response times were significantly higher for accept decisions (one-sided Wilcoxon-Rank-Sum test, W = 1611, p < .001), in accordance with the prediction of the inequity aversion model. In the comparison lesion group, the response times did not differ systematically as a function of decision type (W = 1643, p = .481), favoring the self-gain only model. Note however, that the self-gain-only model is inconsistent with the observed choices per se in these trials in both groups, and therefore does overall not present an appropriate model of our patients’ behavioral data.

**
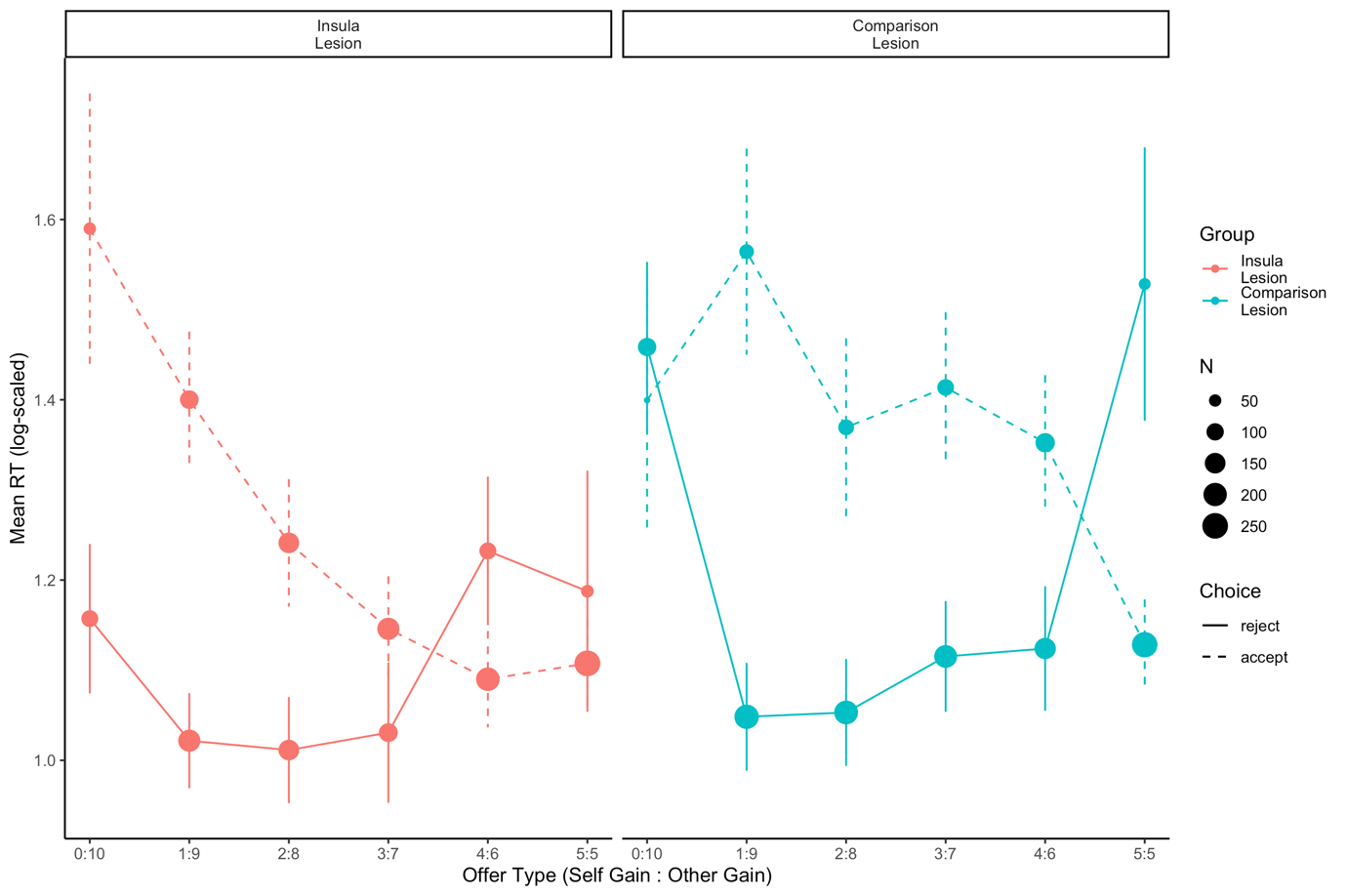
**

**Figure S3: Group-level response times per offer and decision type**

The left panel shows the subjects of the insula lesion group, the right panel shows the subjects of the comparison lesion group. In each panel, dashed lines indicate accept decisions and continuous lines indicate reject decisions. Dots show the group mean, error bars show the standard error of the mean. The size of the dots indicates the number of choices accept or reject decisions respectively.

**Exploration of laterality effects within the insula lesion group**

Within the insula group, n = 24 patients had damage to one insula only (n = 12 to the left, n = 12 to the right). For the remaining six patients, damage to both insulas was observed, however in all cases the extent of damage was much larger (3 to 49 times) on one side than the other, and the lesions on the less affected side were also small in absolute terms. For the exploratory analysis of laterality effects, and for the sample description in the main manuscript, we therefore classified these patients based on the predominately affected insula, resulting in a total n of 16 with damage to the right insula and n of 14 with damage to the left insula.

To test for potential difference in accept/reject choices of patients with (predominantly) right versus left insula damage, a logit-binomial generalized linear mixed effects model (GLME) with lesion laterality, offer (6 levels), and their interaction, as well as volume of insula damage as fixed effects and a subject-level random intercept was conducted in the insula lesion group only. This supplementary explorative analysis confirmed a significant main effect of offer (z = 10.41, p < .0001) and a tentative effect of the extent of insula damage (z = 1.646, p = .099), but found no significant effects of laterality (main effect: z = 0.091, p = .927, laterality x offer interaction: z = -1.021, p = 0.307, see Figure S4).

In summary, reject/accept choices on the UG did not systematically differ between patients with damage to the left versus those to the right insula. Consistently, neuroimaging studies have typically found bilateral activations of the insula during social and non-social decision-making (Studer *et al.*, 2012) and a previous neuropsychological study on risk-sensitive choices likewise found no systematic effects of insula lesion laterality (Clark *et al.*, 2008).


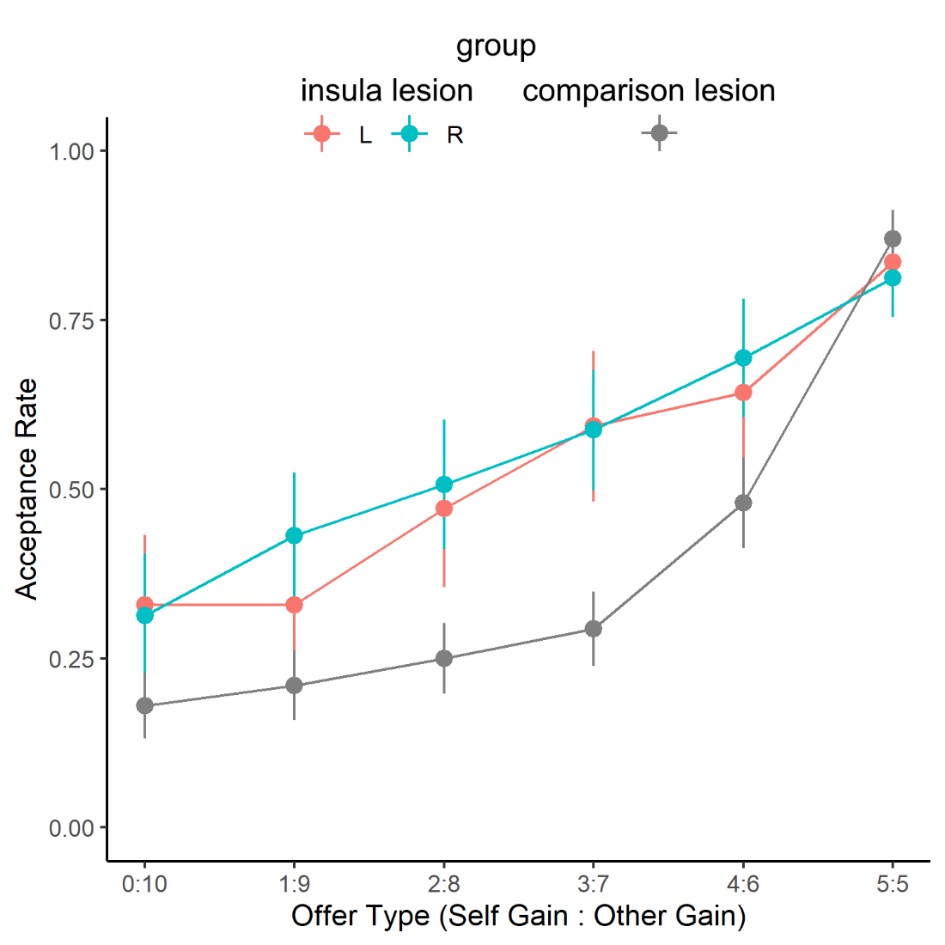


**Figure S4: Responses on the UG as a function of insula lesion laterality**

Average acceptance rates per offer of patients with (predominately) right insula damage (in green) and those with (predominately) left insula damage (in red), and of the lesion comparison group (in grey, plotted for comparison).

**Exploratory lesion-effect mapping within the insula lesion group**

Two sets of exploratory analyses assessed whether the observed effects of insula lesions could be attributed to different subregions within the insula. The first set consistent of voxel-based analyses within the insula, that tested for potential association between damage location and i) average acceptance rates of the disadvantageous offers and ii) model-estimated inequity aversion. Voxels affected in at least 10% of insula lesion patients were considered, and correction for total volume of brain damage and multiple testing (Bonferroni correction) were applied. No voxels superseded the statistical threshold.

In the second set of analyses, we used the six insula subregions defined in the Brainnetome Atlas (Fan *et al.,* 2016), depicted in Figure S5 (see also Figure 4 in Fan *et al.,* 2016 for more information). This atlas was obtained through parcellation based on functional and anatomical connectivity. Noteworthy, and consistent with lesion patterns in stroke patients, this parcellation of insula lesions showed that in the majority of individual patients (67%), lesions spanned at least three subregions, and all six subregions were involved in a third of the sample. Furthermore, each subregion was damaged in more than half of the sample (*dorsal agranular insula*: 57%, *ventral dysgranular and granular insula*: 67%, *dorsal granular insula*: 70%, *dorsal dysgranular insula*: 90%, *hypergranular insula*: 73%), with the exception of the *ventral agranular insula* (40%). Therefore, data separability and a priori-statistical power for detecting potential subregion effects were very limited. Nonetheless, we conducted exploratory regression models assessing whether inequity aversion and average acceptance rates of disadvantageous offers within the insula lesion group were predicted by the extent of damage in each of these six subregions (averaged across left and right hemisphere; 6 predictors, plus additional predictor total lesion volume). No significant effects were observed in either model (inequity aversion: smallest p =.338, *ventral agranular insula*; acceptance rate of disadvantageous offers: smallest p = .202, *dorsal dysgranular insula*).

In summary, we could not identify any differential contributions of insula subregions to the observed effects in the insula lesion group, plausibly due to lack of distinctiveness in the vascular lesions of our stroke sample.


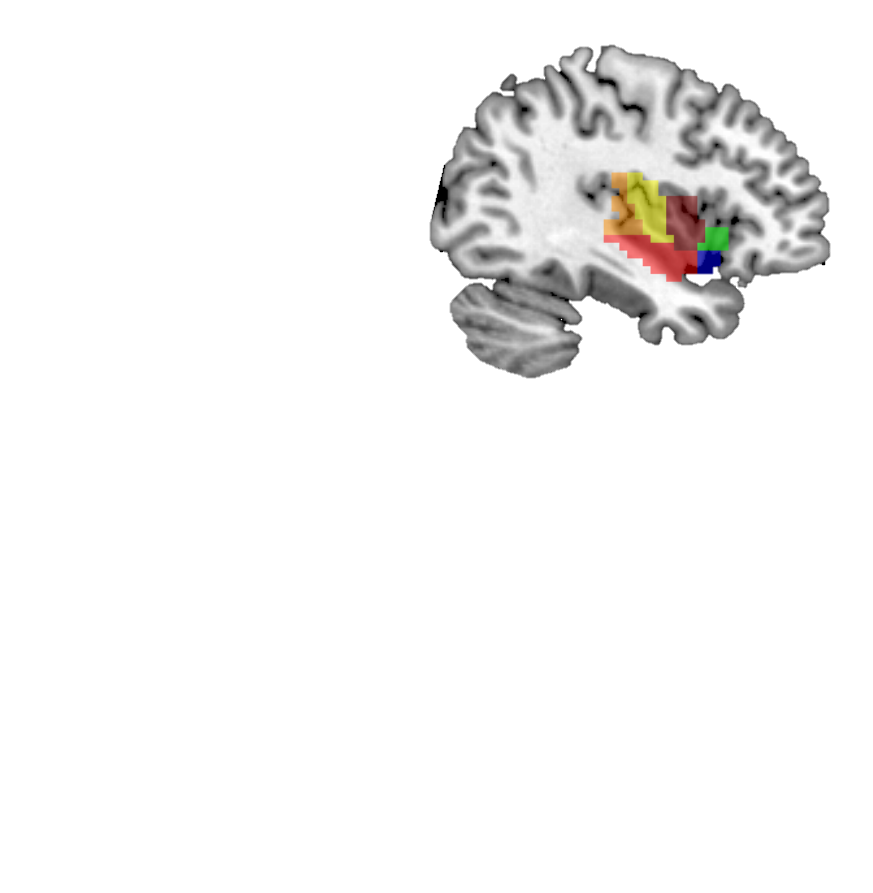


**Figure S5: Insula subregions from the Brainnetome Atlas**

Blue = ventral agranular insula, green = dorsal agranular insula, burgundy = dorsal dysgranular insula, red = ventral dysgranular and granular insula, yellow = dorsal granular insula, orange = hypergranular insula.

**Supplementary analyses on striatal involvement**

Since the insula and dorsal striatum lie within the same vascular territory, damage in our insula lesion group often extended into parts of the dorsal striatum. Post-hoc lesion mapping showed that lesions encroached into the putamen (as defined in the AAL atlas) in n = 26, in n = 11 of which parts of the caudate were also affected; whereas lesions completely spared the dorsal striatum in only n = 4 patients. An ANCOVA comparing the model-estimated inequity aversion in insula lesion patients with (n = 11) and without caudate (n = 19) involvement, controlling for the volume of insula damage and total brain damage, revealed no significant differences between these two subgroups (F = 0.901, p = .351). Due to the small number of patients without putamen involvement, a comparable statistical analysis for the putamen was not possible. Exploratory single-case analysis, however, indicated that inequity aversion was heavily reduced in all four patients without any involvement of the dorsal striatum compared to both the lesion comparison group and the remaining insula lesion group (see Figure S6).

**
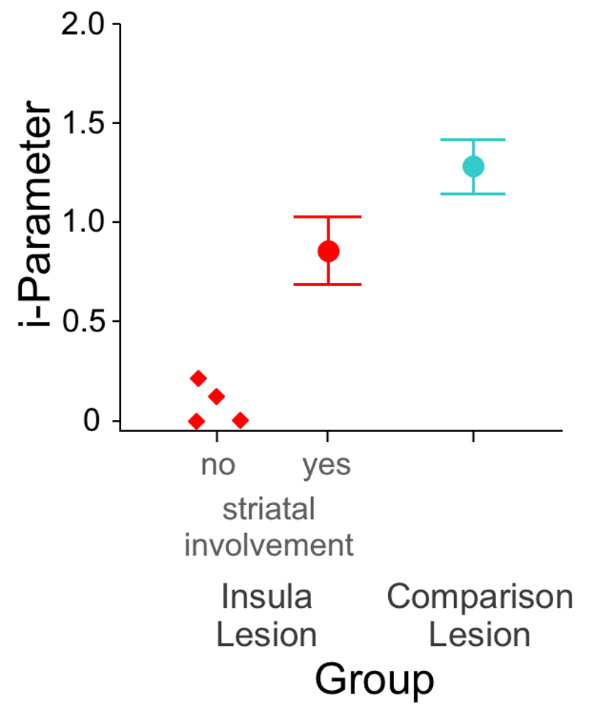
Figure S6: Inequity aversion in insula lesion patients without striatal encroachment**

Model-estimated inequity aversion of the four insula lesion patients without any involvement of the dorsal striatum (red diamonds; individual data points) and average inequity aversion estimates of insula lesion patients with striatal involvement (red circle, averaged data from n=26) and comparison lesion patients (green circle, average data from n=30). Error bars represent SEM.

**Supplementary References**

Clark L, Bechara A, Damasio H, Aitken M, Sahakian B and Robbins T. Differential effects of insular and ventromedial prefrontal cortex lesions on risky decision-making. Brain 2008; 131 (5): 1311-1322.

Fin L, Hai L, Zhuo J, Zhan J, Wang J, *et al.* The Human Brainnetome Atlas: A New Brain Atlas Based on Connectional Architecture. Cerebral Cortex 2016; 26 (8): 3508-26.

Studer B, Apergis-Schoute A, Robbins TW and Clark L. What are the odds? The neural correlates of active choice during gambling. Frontiers in Neuroscience 2012; 6.
